# Case report: dual primary AIDS-defining cancers in an HIV-infected patient receiving antiretroviral therapy: Burkitt’s lymphoma and Kaposi’s sarcoma

**DOI:** 10.1186/s12885-018-5019-9

**Published:** 2018-11-08

**Authors:** Seong Eun Kim, Younggon Jung, Tae Hoon Oh, Uh Jin Kim, Seung-Ji Kang, Hee-Chang Jang, Kyung-Hwa Park, Kyung-Hwa Lee, Sook In Jung

**Affiliations:** 1 Department of Infectious Diseases, Chonnam National University Medical School, 42, Jebong Ro, Donggu, Gwangju, 61469 Korea; 2 Department of Pathology, Chonnam National University Medical School, Gwangju, Republic of Korea

**Keywords:** HIV, Burkitt’s lymphoma, Kaposi’s sarcoma, Antiretroviral therapy

## Abstract

**Background:**

The incidence of AIDS-defining cancers (ADCs) has decreased markedly in the era of highly active antiretroviral therapy (HAART). The occurrence of two ADCs is rare in people living with HIV or AIDS (PWHA) who are severely immunosuppressed or have incomplete virologic suppression.

**Case presentation:**

We report a case of dual primary ADCs, especially NHL followed by KS, in a 70-year-old HIV-infected man who was on antiretroviral therapy and had successful virologic suppression. During HAART, he presented with generalized myalgia and abdominal pain. Multiple liver masses were detected and a biopsy revealed Burkitt’s lymphoma. After three cycles of anticancer chemotherapy with a favorable response, he was diagnosed with cytomegalovirus retinitis and the anti-cancer chemotherapy was discontinued. Despite successful virologic suppression with HAART, human herpes virus-8 associated Kaposi’s sarcoma was diagnosed in his right thigh. He underwent radiation therapy.

**Conclusion:**

These findings suggest that multiple ADCs can occur in PWHA who are receiving HAART and have successful virologic suppression. Healthcare providers caring for PWHA should maintain vigilance for the development of a broad spectrum of cancers.

## Background

The introduction of highly active antiretroviral therapy (HAART) has changed the natural history of opportunistic infections (OIs) and malignancies among people with HIV or AIDS (PWHA). The incidence of AIDS-defining cancers (ADCs), including Kaposi’s sarcoma (KS), non-Hodgkin lymphoma (NHL), and invasive cervical cancer, has decreased significantly [1–3]. Conversely, an increased incidence of non-AIDS related cancers (NADCs) has been reported, especially cancers of the lungs, liver, kidneys, anus, head and neck, and skin, as well as Hodgkin lymphoma [4–7]. ADCs are associated with oncogenic viral infections, including Epstein-Barr virus (EBV), human herpes virus type 8 (HHV-8), cytomegalovirus (CMV), papillomavirus, and possibly other viral agents [3, 8]. The restoration of immune function by HAART may contribute to the decreased incidence of ADCs, which results in fewer AIDS-related deaths.

The occurrence of dual ADCs is rare in the era of antiretroviral therapy. Here, we describe a case of dual primary ADCs in an HIV-infected patient who was receiving HAART and had successful viral suppression.

## Case presentation

A 70-year-old bisexual man was admitted with generalized myalgia and abdominal pain lasting for 7 days. Three months earlier, he was diagnosed with HIV infection during the evaluation of a fever. The initial HIV RNA level was 36,500 copies/mL, with 114 CD4^+^ lymphocytes/μL, which were consistent with the definition of AIDS [9] although the exact timing of HIV infection was unknown. At that time, abdominal and chest computed tomography (CT) showed no abnormality and an ophthalmologic examination showed no evidence of ocular disease. In addition, anti-cytomegalovirus (CMV) IgG was positive. For 3 months, he was treated with an integrase strand transfer inhibitor (INSTI)-based regimen (elvitegravir, cobicistat, tenofovir disoproxil fumarate, and emtricitabine) and showed good adherence.

**Fig. 1:**
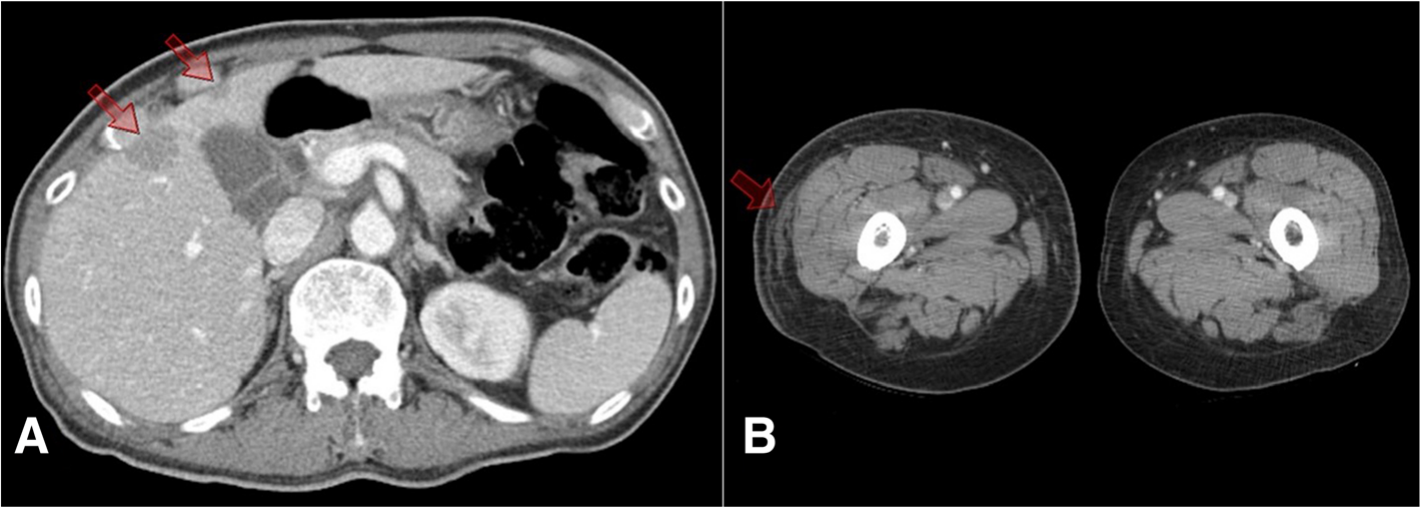
(**a**) CT of the abdomen revealed 2.5- and 1.5-cm low-attenuated lesions in liver segment 5, with other smaller lesions in both hepatic lobes. (**b**) Thigh CT showed edematous changes in subcutaneous tissues of the right thigh

**Fig. 2:**
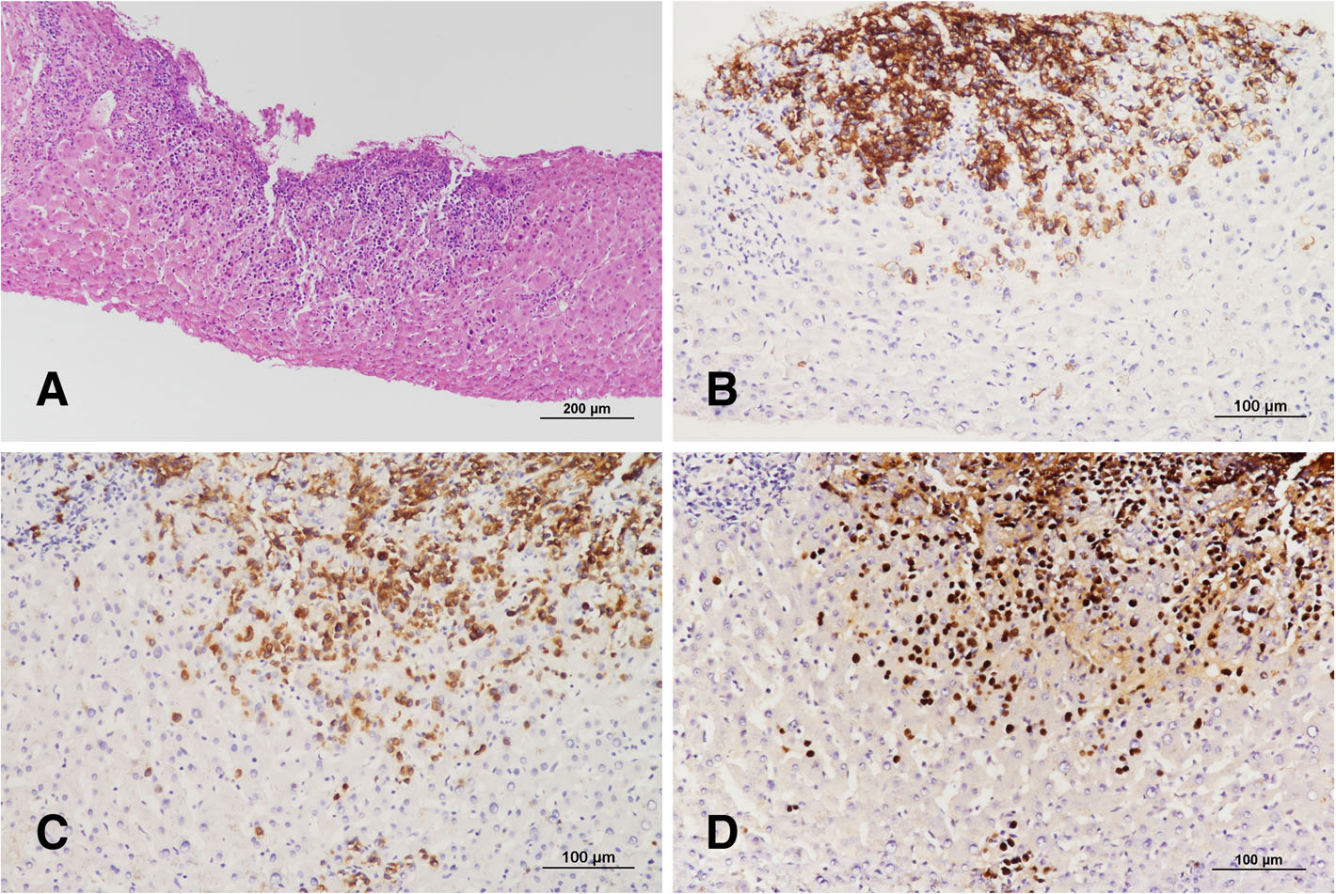
(**a**) Histopathologically, the core biopsy from the liver showed a dense lymphocytic infiltration in the parenchyma (hematoxylin and eosin staining, original magnification × 100). (**b**-**c**) The tumor cells were immunopositive for CD20 (**b**) and CD79a (**c**) (immunohistochemistry, original magnification × 200). (**d**) The tumor cells displayed strong positivity on EBV in situ hybridization, consistent with Burkitt’s lymphoma (in situ hybridization, original magnification × 200)

At the time of the current admission, his vital signs were: blood pressure, 110/80 mmHg; pulse rate, 98/min; body temperature, 38.0 °C; and respiratory rate, 20/min. There was no localized tenderness of the abdomen on physical examination. The laboratory findings showed anemia (hemoglobin, 9.7 g/dL), thrombocytopenia (platelets, 53 × 10^3^/mm^3^), acute kidney injury (creatinine, 1.8 mg/dL), and an elevated lactate dehydrogenase level (LDH; 6608 U/L). No HIV-RNA was detected (< 20 copies/mL), and there were 256 CD4^+^ lymphocytes/μL. Abdominal CT revealed multiple liver masses (Fig. 1a), and a core needle biopsy was performed to differentiate between liver abscess and malignancy. An atypical lymphocytic population composed of medium-sized basophilic cells was observed on hematoxylin and eosin staining (Fig. 2a). Immunohistochemistry was positive for the B cell markers CD20 (Fig. 2b) and CD79a (Fig. 2c), and the Ki-67 labelling index approached 90%. The tumor cells were also positive on EBV in situ hybridization (Fig. 2d). The liver lesion was diagnosed as Burkitt’s lymphoma. An additional diffuse hypermetabolic bone marrow lesion was found on positron emission tomography-CT (PET/CT), and he was confirmed to have stage IV Burkitt’s lymphoma by the Lugano classification.

Three cycles of antineoplastic chemotherapy based on rituximab plus fractionated cyclophosphamide, vincristine, doxorubicin, and dexamethasone (R-hyper-CVAD) were administered, and two cycles of methotrexate were given via an intrathecal route. The HAART was replaced with another INSTI-based regimen (raltegravir, tenofovir disoproxil fumarate, and emtricitabine) after considering potential drug interactions. Interim PET/CT showed a partial response with marked improvement of the lymphomatous involvement in the bone marrow and liver, but hypermetabolic para-aortic, aortocaval lymph nodes remained 3 months after initiating chemotherapy.

Just after the third cycle of R-hyper-CVAD, the patient suddenly complained of decreased visual acuity and an ophthalmologist diagnosed CMV retinitis. At that time, there were 56 CD4^+^ lymphocytes/μL, but no HIV RNA was detected (< 20 copies/mL). Intravitreal and intravenous ganciclovir were used for induction therapy, and valganciclovir maintenance therapy was continued. Chemotherapy was stopped after the third cycle of R-hyper-CVAD due to intolerance and the CMV retinitis. Four months after discontinuing the chemotherapy, PET/CT showed disease progression in the para-aortic, aortocaval lymph nodes, and newly developed lymphomatous involvement was seen in the paravertebral and cardiophrenic space.

**Fig. 3:**
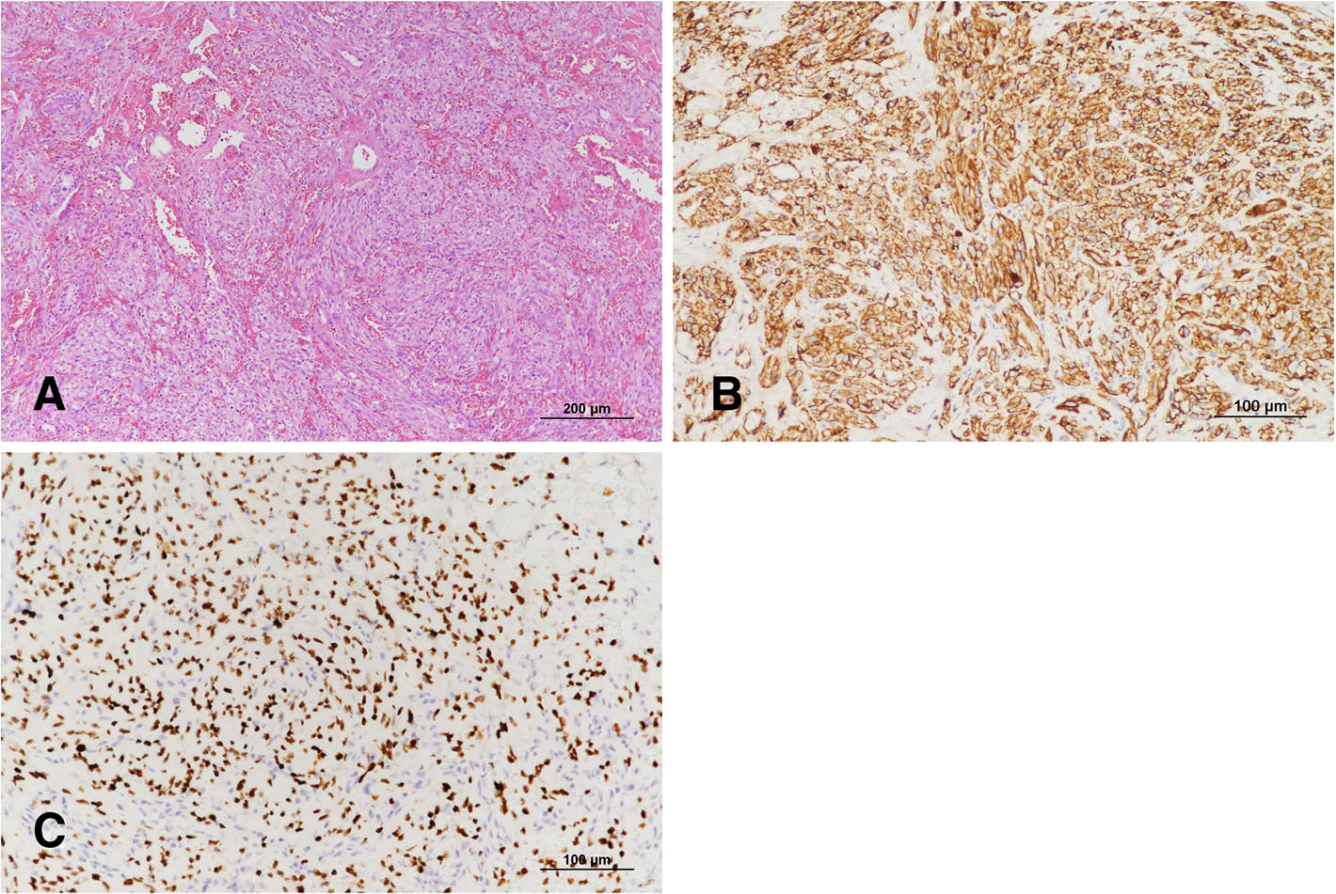
(**a**) The skin biopsy revealed a highly cellular lesion composed of bland spindle cells and intervening irregular vascular spaces (hematoxylin and eosin staining, original magnification × 100). (**b**-**c**) The tumor cells were strongly positive for CD31 (**b**) and HHV-8 LNA-1 (**c**), a diagnostic marker of KS (immunohistochemistry, original magnification × 200)

Ten months after discontinuing systemic chemotherapy, the patient developed swelling and pain in his right thigh. CT showed edematous changes and skin thickening in this area (Fig. 1b, arrow), but there was no remarkable size increase in the inguinal lymph nodes. There were 129 CD4^+^ lymphocytes/μL, and HIV RNA was undetectable. A skin punch biopsy was performed because it was unclear whether the manifestation was related to the Burkitt’s lymphoma. The biopsy specimen was composed predominantly of plump spindle cells with intervening vascular spaces (Fig. 3a). Despite the bland cytology of the tumor cells, frequent mitotic figures were seen and the tumor was infiltrating the dermal collagen fibers. The tumor cells were positive for CD31 (Fig. 3b) and HHV-8 latent nuclear antigen-1 (LNA-1) by immunohistochemistry (Fig. 3c). The histology was consistent with KS. Twenty Grays of radiation were given in five fractions without systemic chemotherapy. Two months after radiation therapy, multiorgan (bone, liver, and pericardium) lymphoma aggravation led to his death.

## Discussion

To our knowledge, this is the first described case of dual primary ADCs (NHL, followed by KS) in an HIV-infected patient who was receiving HAART and had successful viral suppression. Before the era of HAART, opportunistic infections and malignancies were major causes of death in PWHA. The introduction of HAART changed the trend in malignancies in PWHA in many countries [10–12]. The decrease in the prevalence of severe immunodeficiency resulted in a rapid reduction of new ADCs, such as KS and NHL [8, 11, 13, 14]. The Swiss HIV cohort study reported that the incidence of KS and NHL declined from 1,375/10^5^ and 952/10^5^ in the pre-HAART era (1985–1996) to 66.9/10^5^ and 98.4/10^5^, respectively, in the late HAART (2002–2006) period [13]. The incidence of ADCs is higher when patients have lower CD4 cell counts, initially present with cancer or revisit after loss to follow-up [15]. Conversely, the risk of ADC is reduced markedly among patients who remain in care and continue HAART [5]. Coexisting or metachronous KS and NHL are rarely reported in PWHA who have severe immunosuppression or incomplete virologic suppression [16–18]. Furthermore, KS developed first, followed by NHL, or these two ADCs developed simultaneously. Interestingly, the reduction in incidence after the introduction of HAART was pronounced for KS compared with NHL [8]. This suggests that patients cease to be at risk of KS once HAART has improved immune function, although patients with a history of severe immunodeficiency continue to be at risk of NHL despite HAART. In this report, NHL was detected first in a patient who was receiving HAART and showed successful viral suppression; KS developed 13 months later, despite discontinuing systemic chemotherapy for NHL. Dual primary malignancies are not uncommon in the general population. However, the relative risk of multiple ADCs in patients with HIV-related immunodeficiency is unknown. Our findings indicate the possibility of multiple ADCs in PWHA who are receiving HAART and show successful virologic suppression.

HIV-related Burkitt’s lymphoma constitutes 24–35% of all HIV-related NHLs [19]. The cure rate was unsatisfactory in patients with HIV-related NHL before HAART, whereas the introduction of HAART increased the treatment options and improved the outcomes for patients with HIV-related Burkitt’s lymphoma [20]. HAART reduces the decline in CD4 counts during chemotherapy, ameliorating the potential for opportunistic infections. Our patient received HAART continuously during cytotoxic chemotherapy. However, in this patient, chemotherapy for Burkitt’s lymphoma could not be continued because of the occurrence of CMV retinitis despite an excellent response to chemotherapy. CMV reactivation during conventional chemotherapy may occur in a high proportion of patient, resulting in CMV-associated diseases [21]. A number of challenges exist in the management of Burkitt’s lymphoma in PWHA. Along with the high risk of tumor lysis syndrome caused by the high tumor burden, physicians should pay attention to the development of infectious or non-infectious complications during cytotoxic chemotherapy.

HHV-8 infection is necessary for the development of KS [22]. KS was the most common ADC in PWHA, especially homosexual men in the early 1980s [23]. After the introduction of HAART, KS decreased dramatically in both the US and Europe [24]. There are three possible mechanisms by which HAART could block KS. They are HAART-induced immune reconstitution against HHV-8 and infected cells [25, 26], reduced circulating levels of HIV-1 Tat protein and inflammatory cytokines [27–29], and direct anti-spindle cell or anti-angiogenic effects of protease inhibitors [30]. In our patient, KS occurred while he was receiving HAART. KS has recently been reported to occur in people with a well-controlled HIV infection and CD4^+^ T-cell count > 200 cells/mm^3^. A recent study revealed that imperfect HAART adherence and a latest CD4 count < 350 cells/μL were significantly associated with KS development [31]. CD4 lymphocytes are recovered and resulting plateau about 2–3 years after HAART [32]. More frequent incomplete response (< 500/μL) was seen in patients with low CD4 T lymphocyte counts at baseline or long duration of pre-treatment HIV infection [33]. In this patient, initial CD4^+^ lymphocyte count at diagnosis was 114 cells/μL. At the time of KS diagnosis, our patient’s CD4^+^ lymphocyte count was 129 cells/μL although no HIV RNA was detected. The risk for subsequent KS among NHL survivors is relatively high in the general population [34]. Although the risk for the development of KS in HIV-positive NHL survivors is not known, the damaged immune system caused by HIV infection, NHL progression, and cytotoxic chemotherapy might have contributed to the development of KS in this patient.

Tests determining the risk of opportunistic infections should be performed for every patient with HIV entering into care as recommended in HIV primary care and opportunitstic infection guidelines [35, 36]. CMV retinitis can be diagnosed through a dilated pupil during an ophthalmoscopic examination by an experienced ophthalmologist. However, blood tests including CMV antigen detection, culture, or PCR are not recommended for diagnosis of CMV end-organ disease because of their poor positive predictive values. Serum antibody to CMV is not diagnostically useful [36]. In addition, use of serologic testing for HHV-8 antibodies is currently not indicated for either diagnostic testing or routine screening for HHV-8-related illnesses due to lack of standardization and poor sensitivity and specificity [37]. Although HIV-infected patients are more likely to have detectable plasma EBV DNA than are HIV-uninfected control subjects, the absolute level of EBV DNA is not predictive of progression to AIDS-related NHL [38]. In this patient, serum IgG antibody to CMV was detected at the time of HIV diagnosis. However, ophthalmologic examination revealed no evidence of CMV retinitis. Chest and abdominal CT showed no abnormality initially. Eventually, dual primary ADCs and CMV retinitis occurred despite viral suppression. Clinicians continue to monitor ADCs and OIs even in the situation of successful viral suppression with ART. Furthermore, development of diagnostic tools to easily predict ADCs and OIs is needed.

## Conclusion

In summary, dual primary ADCs, especially NHL followed by KS, are uncommon in PWHA who are receiving HAART and show successful virologic suppression. Healthcare providers caring for PWHA should maintain high vigilance for the development of a broad spectrum of cancers in all HIV/AIDS patients.

## Abbreviations

ADCs: AIDS defining cancers
CD: Cluster of differentiation
CMV: Cytomegalovirus
CT: Computed tomography
EBV: Epstein-Barr virus
HAART: Highly active antiretroviral therapy
HHV-8: Human herpes virus type 8
INSTI: Integrase strand transfer inhibitor
KS: Kaposi’s sarcoma
LDH: Lactate dehydrogenase
LNA-1: Latent nuclear antigen-1
NADC: Non-AIDS related cancers
NHL: Non-Hodgkin’s lymphoma
OI: Opportunistic infection
PET-CT: Positron emission tomography-computed tomography
PWHA: People with HIV or AIDS
R-hyper-CVAD: Rituximab plus fractionated cyclophosphamide, vincristine, doxorubicin, and dexamethasone
